# Correlation between SMADs and Colorectal Cancer Expression, Prognosis, and Immune Infiltrates

**DOI:** 10.1155/2023/8414040

**Published:** 2023-03-08

**Authors:** Ning Ding, Hongbiao Luo, Tao Zhang, Tianshu Peng, Yanru Yao, Yongheng He

**Affiliations:** ^1^Hunan University of Chinese Medicine, Changsha, Hunan 410208, China; ^2^Department of Anorectal Surgery, Chenzhou NO. 1 People's Hospital, Chenzhou 423000, China; ^3^Department of Anorectal Surgery, The Second Affiliated Hospital of Hunan University of Chinese Medicine, Changsha, Hunan 410005, China; ^4^Department of Anorectal Surgery, The Affiliated Hospital of Hunan Academy of Traditional Chinese Medicine, Changsha, Hunan 410006, China

## Abstract

**Background:**

In recent years, the incidence and mortality of colorectal cancer (CRC) are increasing, and the 5-year survival rate of advanced metastatic CRC is poor. Small mothers against decapentaplegic (SMAD) superfamily are intracellular signal transduction proteins associated with the development and prognosis of a variety of tumors. At present, no study has systematically analysed the relationship between SMADs and CRC.

**Methods:**

Here, R3.6.3 was used to analyse the expression of SMADs in pan-cancer and CRC. Protein expression of SMADs were analysed by Human Protein Atlas (HPA). Gene expression profiling interactive analysis (GEPIA) was used to evaluate the correlation between SMADs and tumor stage in CRC. The effect of R language and GEPIA on prognosis was analysed. Mutation rates of SMADs in CRC were determined by cBioPortal, and potentially related genes were predicted using GeneMANIA. R analysis was used to correlate immune cell infiltration in CRC.

**Results:**

Both SMAD1 and SMAD2 were found to be weakly expressed in CRC and correlated with the immune invasion level. SMAD1 was correlated with patient prognosis, and SMAD2 was correlated with tumor stage. SMAD3, SMAD4, and SMAD7 were all expressed at low levels in CRC and associated with a variety of immune cells. SMAD3 and SMAD4 proteins were also expressed at low levels, and SMAD4 had the highest mutation rate. SMAD5 and SMAD6 were overexpressed in CRC, and SMAD6 was also associated with patient overall survival (OS) and CD8+ T cells, macrophages, and neutrophils.

**Conclusions:**

Our results reveal innovative and strong evidence that SMADs can be used as biomarkers for the treatment and prognosis of CRC.

## 1. Introduction

Colorectal cancer (CRC) is widely known as one of the most pervasive malignancies due to its third highest morbidity (10.0%) and second highest mortality (9.4%) among all cancers worldwide, and its morbidity and mortality are on the rise year by year [1]. The 5-year survival rate for advanced metastatic colorectal cancer is less than 20% [2]. The main treatment methods for CRC are surgery, radiotherapy, and chemotherapy, which are good for early colorectal cancer but poor for advanced and metastatic CRC [3]. There is no good treatment for advanced metastatic colorectal cancer. To eliminate the high incidence and mortality of CRC, further exploration of meaningful biomarkers is urgently needed to strengthen its therapeutic efficacy.

There are eight small mothers against decapentaplegic (SMAD) codes in the human genome [4]. SMAD proteins are a family of signal transduction molecules involved in the transforming growth factor *β* (TGF-*β*) ligand pathway. SMADs belong to the intracellular protein family with a total length of 500 amino acids, among which SMAD1, SMAD2, SMAD3, SMAD5, and SMAD8 act as TGF-*β* receptors in mammals, of which SMAD8 is generally expressed as SMAD9. SMAD4 is a common pathway mediator, and SMAD6 and SMAD7 inhibit SMAD [5]. The main function of SMADs is to control the gene program, transcriptional regulation, and signal transduction, which can mediate TGF-*β*/SMAD, Notch, ERK (extracellular regulated protein kinases)/MAPK (mitogen-activated protein kinase), Hippo, JAK (janus kinase)/STAT (signal transducer and activator of transcription), Hedgehog, BMP (bone morphogenetic protein)/SMAD, and so on [6]. SMADs have been implicated in cell proliferation, migration, apoptosis, and immune regulation of cancer cells [7–9]. SMADs are associated with lung, pancreas, liver, gastrointestinal tumors, and so on [10]. However, there are few comprehensive studies on the expression, prognosis, and immune infiltration of the SMAD superfamily as a whole and colorectal cancer.

With the wide application of big data sets, the collection in the field of biomedicine is called omics, including various genomics, transcriptomics, proteomics, and metabolomics, from this perspective, many new and better ways of disease diagnosis and treatment and mechanism research have been found [11]. Omics methods have been applied in the screening and diagnosis of various tumors, including CRC. The application of various omics methods is of great value in understanding the pathological process of CRC, identifying CRC markers and predicting prognosis [12].

In this study, we used public databases and R language for in-depth analysis of the correlation between SMADs and the occurrence and development of CRC, as well as prognostic analysis and immune infiltration analysis of CRC patients to demonstrate the value of different SMADs in the occurrence, prognosis, and immune infiltration of colorectal cancer.

## 2. Materials and Methods

### 2.1. The Human Protein Atlas (HPA)

The Human Protein Atlas (HPA) (http://www.proteinatlas.org/pathology) maps human proteins by analysing the effects of clinical results on various omics, primarily based on the relationship between the genome-wide transcriptome of protein-coding genes of 17 cancer types and clinical results [13]. In this study, we used this database to investigate the relationship between SMAD proteins and CRC.

### 2.2. The Gene Expression Profiling Interactive Analysis (GEPIA)

GEPIA (http://gepia.cancer-pku.cn/) is an online web address based on The Cancer Genome Atlas (TCGA) and the Genotype-Tissue Expression database (GTEx) consisting of thousands of tumor and healthy tissue sample data using standard processing pipelines, providing key interactive and customizable functionality [14]. In this study, GEPIA was used to analyse the correlation between SMADs and the pathological stage of CRC, and its prognostic value was analysed by this method.

### 2.3. cBioPortal

cBioPortal (http://cbioportal.org) is a free open platform for multidimensional cancer genome analysis, detection, and visualization at the deoxyribonucleic acid (DNA) level [15]. In this study, cBioPortal was used to predict mutation rates of the SMAD gene family in CRC.

### 2.4. GeneMANIA

GeneMANIA (http://www.genemania.org) is a rich and friendly website for hypothesis of gene function, analysis of gene lists, identification of functionally similar genes, biofunctional genomics, and more [16]. In this study, we explored the SMAD interaction network and associated genes through the GeneMANIA database.

### 2.5. Statistical Analysis

All statistical analyses were performed using R (V3.6.3). The differences were visualized using the ggplot2 software package. Paired *t* tests and Mann–Whitney *U* tests were used to detect differences between colorectal cancer tissues and adjacent normal tissues. The R package survminer was used for visualization of prognostic value, and the survival software package was used for statistical analysis of survival data. The single sample gene enrichment analysis (ssGSEA) package of gene set variation analysis (GSVA) [17] was used for immune infiltration analysis, and the Shapiro–Wilk normality test and Spearman correlation coefficient calculation were used to detect the correlation of immune infiltration.

## 3. Results

### 3.1. Differential Expression of SMADs in Pan-Cancer and CRC

**Figure 1:**
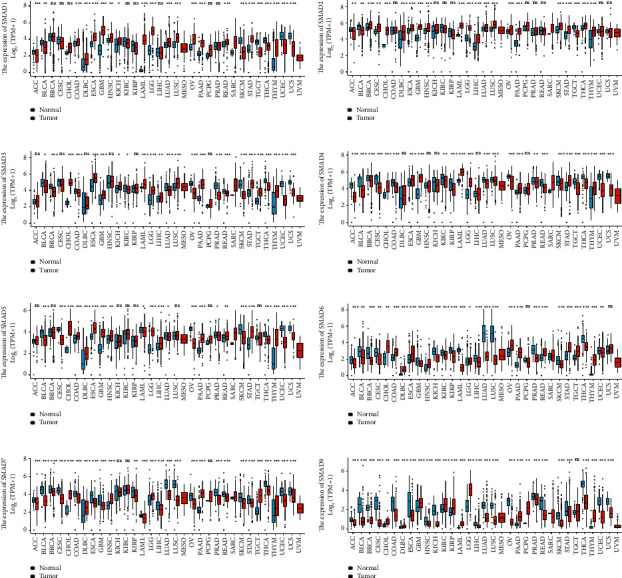
Differential expression of SMADs in pan-cancer. (a) SMAD1, (b) SMAD2, (c) SMAD3, (d) SMAD4, (e) SMAD5, (f) SMAD6, (g) SMAD7, and (h) SMAD9.

**Figure 2:**
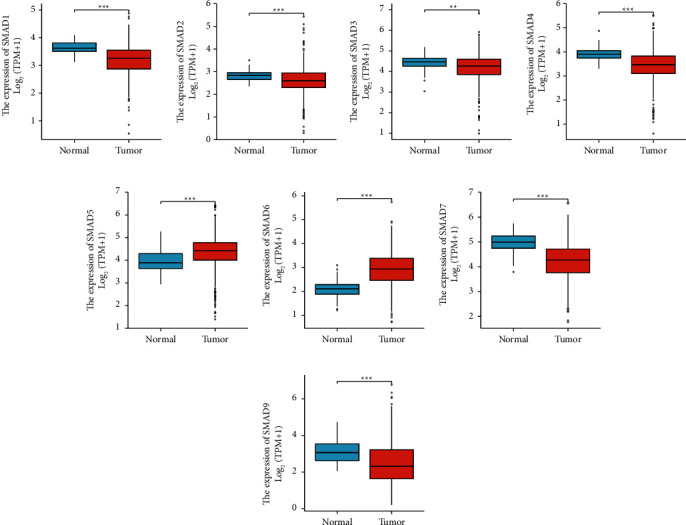
Differential expression of SMADs in colorectal cancer. Compared with normal tissue, (a) SMAD1, (b) SMAD2, (c) SMAD3, (d) SMAD4, (g) SMAD7, and (h) SMAD9 were significantly upregulated. (e) SMAD5 and (f) SMAD6 were significantly downregulated.

The expression differences of SMADs across cancers were detected by the ggplot2 software package, as shown in Figure 1. Then, the same package was used to detect the differential expression of SMADs in 51 normal samples and 647 colorectal cancer samples (Figure 2), and the results showed that the expression levels of SMAD1-4, SMAD7, and SMAD9 were significantly downregulated, while the expression levels of SMAD5 and SMAD6 were significantly upregulated. The specific situation was analysed as follows.

Unpaired sample analysis showed that the expression of SMAD1 in CRC was significantly lower than that in adjacent colorectal normal tissues (Figure 2(a), 3.207 ± 0.539 vs. 3.654 ± 0.236, *p* < 0.001), the expression of SMAD2 in CRC was absolutely lower than that in adjacent colorectal normal tissues (Figure 2(b), 2.604 ± 0.591 vs. 2.823 ± 0.246, *p* < 0.001), the expression of SMAD3 in CRC was significantly lower than that in adjacent colorectal normal tissues (Figure 2(c), 4.197 ± 0.650 vs. 4.426 ± 0.384, *p* = 0.002), the expression of SMAD4 in CRC was absolutely lower than that in adjacent colorectal normal tissues (Figure 2(d), 3.440 ± 0.644 vs. 3.908 ± 0.288, *p* < 0.001), the expression of SMAD7 in CRC was significantly lower than that in adjacent colorectal normal tissues (Figure 2(g), 4.225 ± 0.706 vs. 4.990 ± 0.423, *p* < 0.001), and the expression of SMAD9 in CRC was absolutely lower than that in adjacent colorectal normal tissues (Figure 2(h), 2.486 ± 1.164 vs. 3.125 ± 0.625, *p* < 0.001). The expression levels of SMAD5 (Figure 2(e)) and SMAD6 (Figure 2(f)) in CRC tissues were significantly higher than those in adjacent normal colorectal tissues (*p* < 0.001), and the statistical results were 4.364 ± 0.723 vs. 3.952 ± 0.446 and 2.916 ± 0.64 vs. 2.085 ± 0.394, respectively.

### 3.2. Correlation between SMADs and CRC Tumor Stage

**Figure 3:**
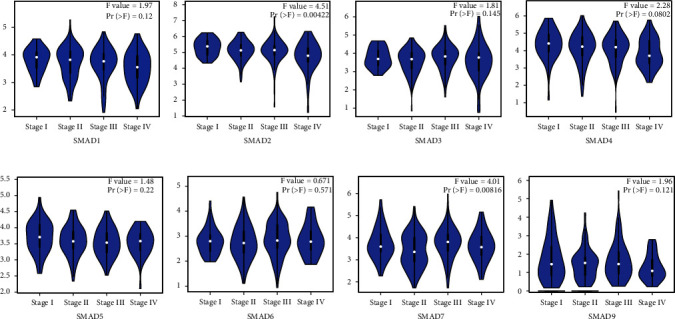
Correlation between SMAD expression and tumor stage in CRC patients (GEPIA). (a) SMAD1, (c) SMAD3, (d) SMAD4, (e) SMAD5, (f) SMAD6, and (h) SMAD9. (b) SMAD2 and (g) SMAD7 were significantly correlated with tumor staging (*P* < 0.05).

By evaluating the correlation between SMAD expression and tumor stage in CRC patients, the results are shown in Figure 3. The analysis results showed that the SMAD2 and SMAD7 groups had noticeable differences (Figures 3(b) and 3(g), all *p* < 0.05), while SMAD1, SMAD3, SMAD4, SMAD 5, SMAD6, and SMAD9 groups had no significant differences (Figure 3(a), Figures 3(c)–3(h), all *p* > 0.05).

### 3.3. Protein Expression of SMADs in CRC

**Figure 4:**
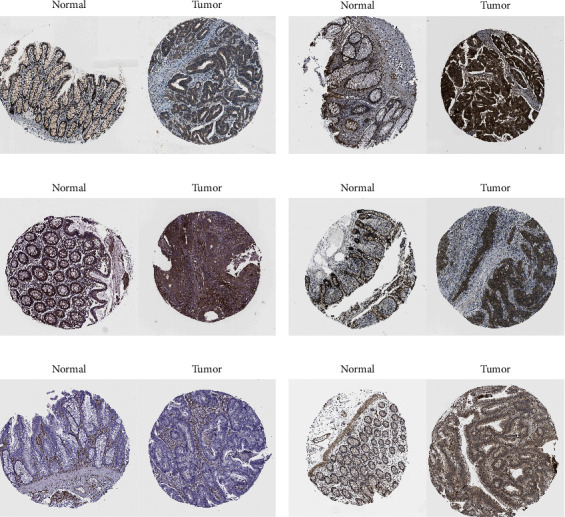
SMADs protein levels in CRC patients. (a) SMAD1 and (b) SMAD2 were highly expressed. Low expression of (c) SMAD3, (d) SMAD4, (e) SMAD5, and (f) SMAD6 were constant.

Protein expression of SMADs in normal intestine and CRC tissues was analysed by HPA, as shown in Figure 4. The results showed that the protein expression levels of SMAD1 and SMAD2 were significantly increased in CRC tissues (Figures 4(a) and 4(b)), the protein expression levels of SMAD3, SMAD4, and SMAD5 were significantly decreased in CRC tissues (Figures 4(c)–4(e)), and the protein expression levels of SMAD7 was not significantly different (Figure 4(f)).

### 3.4. Prognostic Value of SMADs in Colorectal Cancer

**Figure 5:**
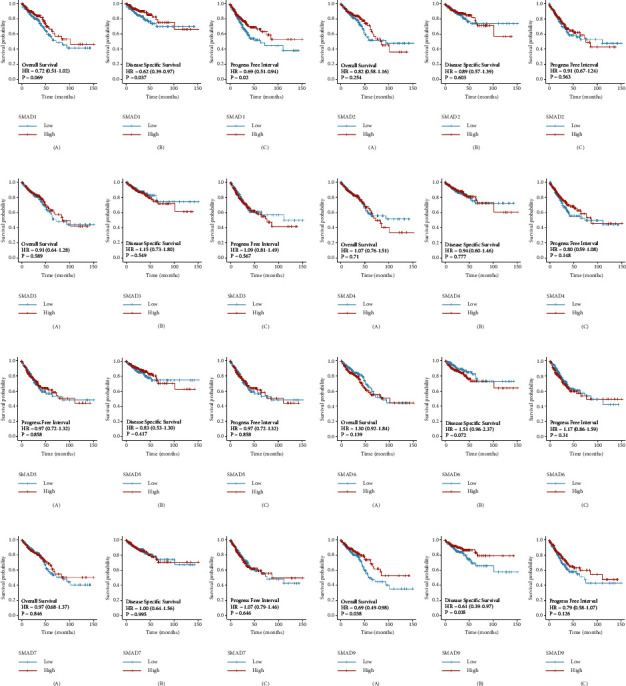
R language analysis of the prognostic value of SMADs in CRC patients. (a) SMAD1 in CRC patients was associated with DSS (*p* = 0.037) and PFI (*p* = 0.02), and (h) SMAD9 was associated with OS (*p* = 0.038) and DSS (*p* = 0.035). (b) The OS, DSS, and DFI of SMAD2 in CRC patients. (c) The OS, DSS, and DFI of SMAD3 in CRC patients. (d) The OS, DSS, and DFI of SMAD4 in CRC patients. (e) The OS, DSS, and DFI of SMAD5 in CRC patients. (f) The OS, DSS, and DFI of SMAD6 in CRC patients. (g) The OS, DSS, and DFI of SMAD7 in CRC patients.

R package survminer and survival were used to analyse overall survival (OS), disease-specific survival (DSS), and progression-free interval (PFI) indicators of survival prognosis of CRC patients by SMADs, as shown in Figure 5. The results showed that SMAD1 was significantly correlated with DSS (*p* = 0.037) and PFI (*p* = 0.02) in CRC patients (Figure 5(a)). SMAD9 was significantly correlated with OS (*p* = 0.038) and DSS (*p* = 0.035) in CRC patients (Figure 5(h)), while other results showed no significant differences.

**Figure 6:**
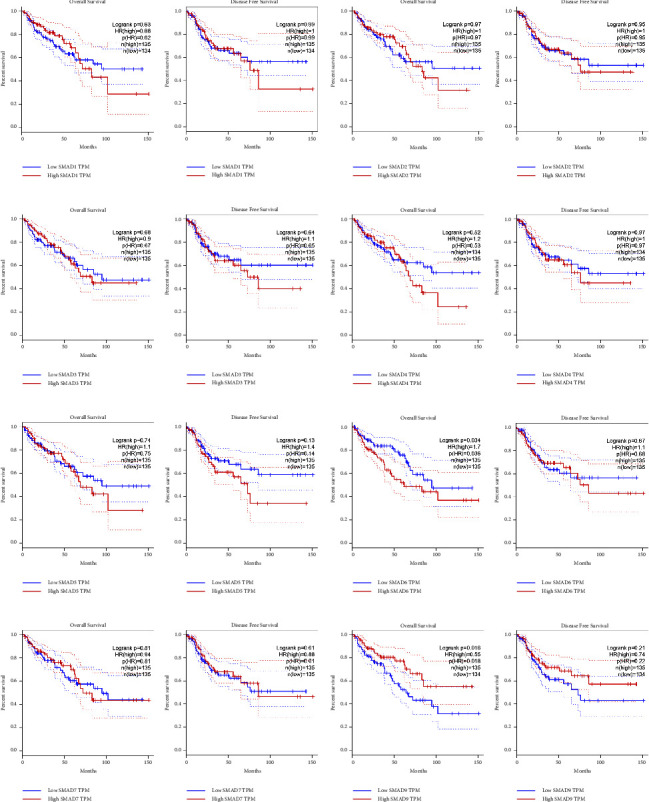
Survival and prognosis analysis of SMADs in CRC patients (GEPIA). (f) SMAD6 and (h) SMAD9 were related to OS (*p* = 0.034, *p* = 0.016). (a) The OS and DFS of SMAD1 in CRC patients by GEPIA. (b) The OS and DFS of SMAD2 in CRC patients by GEPIA. (c) The OS and DFS of SMAD3 in CRC patients by GEPIA. (d) The OS and DFS of SMAD4 in CRC patients by GEPIA. (e) The OS and DFS of SMAD5 in CRC patients by GEPIA. (g) The OS and DFS of SMAD7 in CRC patients by GEPIA.

GEPIA was used to analyse OS and disease-free survival (RFS) indicators of the prognostic value of SMADs for CRC patients, as shown in Figure 6. Analysis showed that SMAD6 and SMAD9 were significantly correlated with OS in CRC patients (Figures 6(f) and 6(h)), while no significant correlations were found in others.

### 3.5. Analysis of SMAD Gene Mutation and Interaction Expression in CRC

**Figure 7:**
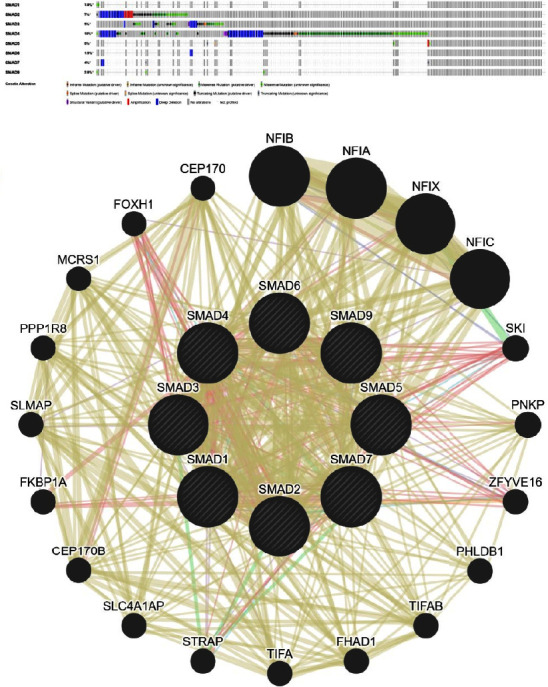
Mutation and expression analysis of SMAD genes in CRC (cBioPortal and GeneMANIA). (a) Summary of SMADs mutations. (b) SMAD-related proteins and interaction networks.

The frequency of SMAD changes in CRC was detected by cBioPortal. The results showed that in 881 CRC patients, the mutations of SMAD1 and SMAD6 were 1.9%, SMAD2 was 7%, SMAD3 and SMAD5 were 5%, SMAD4 was 18%, and the mutation rate was 4% for SMAD7 and 2.8% for SMAD9. The OncoPrints contained in-frame mutations, missense mutations, splice mutations, truncating mutations, structural variants, amplifications, deep deletions, and no alterations (Figure 7(a)). Through the GeneMANIA database, twenty genes associated with the interaction network with SMADs were analysed (Figure 7(b)).

### 3.6. Correlation with Immune Infiltration

**Figure 8:**
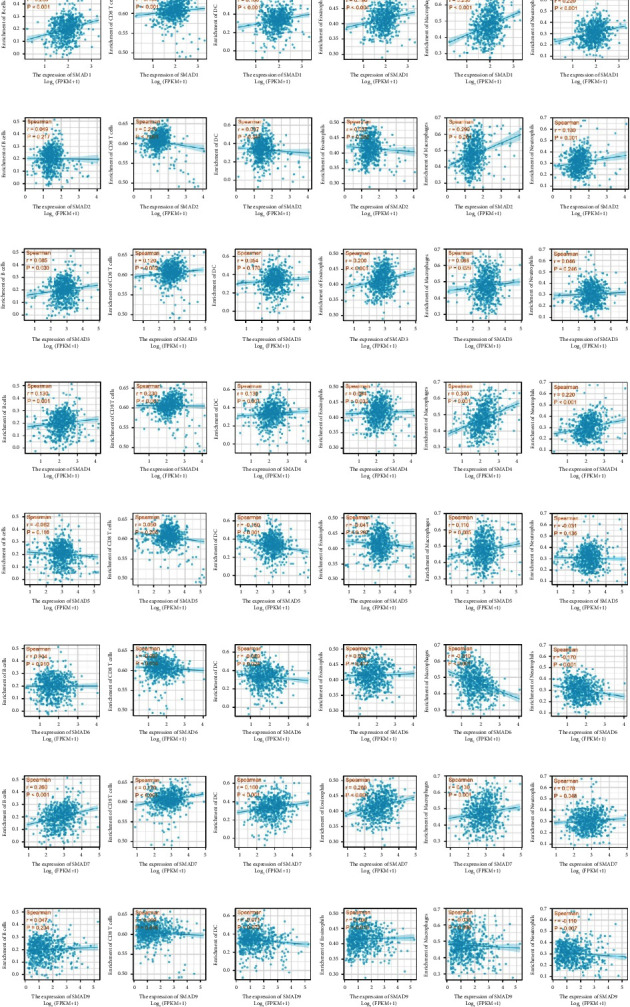
R language was used to analyse the correlation between the differentially expressed SMADs and immune cell infiltration. The expression of (a) SMAD1, (d) SMAD4, and (g) SMAD7 was positively correlated with the infiltration of B cells, CD8+ T cells, dendritic cells, eosinophils, macrophages, and neutrophils. (b) SMAD2 was positively correlated with the infiltration of CD8+ T cells, macrophages, and neutrophils. (c) SMAD3 was positively correlated with the infiltration of B cells, CD8+ T cells, eosinophils, and macrophages. (e) SMAD5 was negatively correlated with dendritic cells and positively correlated with macrophages. (f) SMAD6 was positively correlated with dendritic cells and negatively correlated with CD8+ T cells, macrophages, and neutrophils. (h) SMAD9 was positively correlated with eosinophils and negatively correlated with neutrophils.

The ssGSEA package of GSVA was used to comprehensively analyse the relationship between SMADs and immune cell infiltration, as shown in Figure 8 and Table 1. The results showed that the expression of SMAD1, SMAD4, and SMAD7 was positively correlated with the infiltration of B cells, CD8+ T cells, dendritic cells (DCs), eosinophil macrophages, and neutrophils (Figures 8(a), 8(d), and 8(g)). SMAD2 expression was positively correlated with CD8+ T cells, macrophages, and neutrophils (Figure 8(b)). SMAD3 expression was positively correlated with B cells, CD8+ T cells, eosinophils, and macrophages (Figure 8(c)). SMAD5 expression was positively correlated with macrophage infiltration, while SMAD5 expression was negatively correlated with DC infiltration (Figure 8(e)). SMAD6 expression was positively correlated with DC infiltration, and SMAD6 expression was negatively correlated with CD8+ T cell, macrophage, and neutrophil infiltration (Figure 8(f)). The expression of SMAD9 was positively correlated with eosinophil infiltration, and the expression of SMAD9 was negatively correlated with neutrophil infiltration (Figure 8(h)).

## 4. Discussion

Studies have shown that SMADs are involved in the development, metastasis, prognosis, and immune microenvironment of many tumors. Immune infiltrating cells are related to the tumor microenvironment and influence tumor growth and metastasis. The high expression of SMAD1, SMAD2, and SMAD4 in gastric cancer tissues is significantly correlated with the prognosis of patients [18]. Studies related to lung cancer have found that the expressions of SMAD6, SMAD7, and SMAD9 in SMADs are downregulated in lung cancer and significantly correlated with the prognosis of patients [19]. However, studies related to SMADs and the occurrence, development, prognosis, and immunity of CRC have not been fully clarified.

SMAD1 is the activation type of SMAD receptor, which is involved in modifying cell growth, differentiation, apoptosis, and other processes and plays an important role in the body's immune system. Current studies on SMAD1 in CRC have shown that high expression of SMAD1 can induce apoptosis of CRC [20]. SMAD1 can promote the occurrence of CRC tumors and induce migration and autophagy processes [21]. This study claimed that low expression of SMAD1 in colorectal cancer was related to prognosis and immune cell infiltration, but SMAD1 protein was significantly increased in colorectal cancer tissues. These results suggest that high SMAD1 expression can be used as a diagnostic marker for CRC and as a marker associated with poor prognosis and immunoinfiltration when SMAD1 begins to be low expressed in CRC.

SMAD2 plays different roles in different stages of cancer by regulating various biological processes [22]. In colorectal cancer, the tumor suppressor gene NIT1 is realized by activating the SMAD2/3 signaling pathway [23]. SMAD2 can promote the development of CRC by regulating the polarization of tumor macrophages [24]. In this study, SMAD2 expression in CRC was low, which was significantly different from colorectal cancer tumor stage, associated with CD8+ T cells, macrophages, and neutrophils, and had a high mutation rate. The results of this study are consistent with those of other studies, suggesting that low expression of SMAD2 is correlated with clinical malignancy and affects tumor immune microenvironment.

SMAD3 plays the dual role of oncogene and tumor suppressor gene in tumor formation, and can be used as a prognostic marker for tumors [22]. SMAD4 is a tumor suppressor gene that plays a central role in TGF-*β* signaling pathway transduction [25]. In CRC, SMAD3 reduces its expression through miR-4429 and ultimately inhibits the occurrence, development, and metastasis of cancer cells [26]. A meta-analysis showed that a high mutation rate of SMAD4 in CRC patients was associated with poor prognosis but not with clinical stage [27]. This study showed that SMAD3, SMAD4, and their proteins were significantly underexpressed in colorectal cancer. However, there was no significant correlation between tumor stage and prognosis. The maximum mutation rate of SMAD4 in CRC was 18%. Studies on immune infiltration have shown that SMAD3 and SMAD4 are associated with a variety of immune cells. Our results are generally consistent with previous reports, suggesting that SMAD3 and SMAD4 can act as tumor suppressor genes of CRC and influence patient immune status. However, whether SMAD4 can be used as a prognostic indicator needs further validation.

SMAD5 mediates TGF-*β* superfamily ligand signaling pathways as oncogenic genes [28]. SMAD6 can also regulate TGF-*β* signaling pathway, which is conducive to tumor growth, spread, and metastasis [29]. Overexpression of miR-186-5p in CRC can significantly reduce SMAD6, ultimately inhibiting the proliferation and migration of CRC cells and increasing the apoptosis of CRC cells [30]. This study found that SMAD5 and SMAD6 were significantly overexpressed in colorectal cancer. SMAD6 was significantly correlated with OS. These results are consistent with our study of SMAD5 and SMAD6. These results demonstrated that SMAD5 and SMAD6 could be used as oncogenes of CRC, and SMAD6 could also be used as a prognostic biomolecule.

SMAD7 is an inhibitor of TGF-*β* signaling pathway and antagonizes TGF-*β*-mediated diseases. SMAD7 plays a dual role in different tumor stages. As a tumor suppressor gene in the early stage and a tumor promoter gene in the late stage, SmAD7 is positively correlated with the degree of malignancy [31]. In CRC, SMAD7 can upregulate miR-424 by silencing circTBL1XR1, thus promoting the proliferation, invasion, and metastasis of CRC [32]. miR-4775 overexpression in CRC promotes invasion, metastasis, and epithelial-mesenchymal transition (EMT) processes of cancer cells by activating SMAD7 [33]. In this study, SMAD7 expression was significantly reduced in CRC and was associated with a variety of immune cells. Our study is consistent with the current relevant experimental verification, and the current literature suggests that there is a difference in colorectal-related expression between this study and SMAD7. Considering the dual role of SMAD7, CRC tissues may be in different stages, which is consistent with the actual situation. SMAD7 is both an oncogene and a tumor suppressor gene in CRC and can be used as a marker to evaluate the state of the immune microenvironment.

However, there are only eight members of the SMAD family from 1 to 8. However, some databases SMAD8 is directly named SMAD9, and some databases have both SMAD8 and SMAD9, so it is impossible to perform specific analysis, so further analysis will not be conducted here.

Our study has some shortcomings. First, this study was mainly obtained through database analysis without relevant experimental verification. To better study the relationship between CRC and SMADs, experimental verification is needed to further verify the results and make the results more convincing. Second, due to the ambiguity between SMAD8 and SMAD9 in different databases, specific analysis is not possible. Therefore, our team needs to continue to carry out relevant experimental verification in cell, animal, and clinical aspects.

## 5. Conclusions

In conclusion, this study used R language and several different database systems to analyse the differential expression, mutation rate, prognostic analysis, and immune infiltration of SMAD family members in CRC. The results showed that SMAD1, SMAD2, SMAD3, SMAD4, and SMAD7 were significantly downregulated in CRC, while SMAD5 and SMAD were significantly upregulated in CRC. SMAD1 and SMAD2 proteins were significantly increased in CRC, SMAD3, SMAD4, and SMAD5 proteins were significantly decreased in CRC, and SMAD7 and SMAD9 protein expression was not significantly different. Only SMAD2 was associated with tumor stage of CRC. In terms of prognostic analysis, only SMAD1 was significantly correlated with DSS and PFI, while SMAD6 was significantly correlated with OS. SMAD4 had the highest mutation rate. In immune infiltration, SMAD1, SMAD2, SMAD3, SMAD4, and SMAD7 were positively correlated with a variety of immune cells. By studying the relationship between SMADs family and CRC, in clinical practice, patients with high expression of SMAD1 and SMAD2 and low expression of SMAD3, SMAD4, and SMAD5 in tissue specimens can be identified as CRC, which can be used as diagnostic markers. In order to understand the stage of the tumor, the increase of SMAD2 value can be detected. Based on the correlation between the expression level of a large number of patients and the stage, the interval range can be formulated to further determine the malignant degree of CRC in clinic. The high expression of SMAD1 and low expression of SMAD6 can be detected to determine the prognosis of patients. In order to understand the immune microenvironment of CRC and develop immunotherapy methods, SMAD1, SMAD2, SMAD3, SMAD4, and SMAD7 of patients are of guiding significance. Through the above systematic discussion, the diagnosis, treatment, and survival prognosis of CRC patients can be evaluated clinically by detecting the expression level of SMADs family, which is convenient and has guiding value.

## Figures and Tables

**Table 1:** Correlation between immune cell infiltration and SMADs in CRC.

Genes	Spearman	*Cells*					
		B cells	CD8+ T cells	DC	Eosinophils	Macrophages	Neutrophils
SMAD1	r	0.230	0.190	0.160	0.190	0.280	0.220
	p	<0.001	<0.001	<0.001	<0.001	<0.001	<0.001

SMAD2	r	0.049	0.210	0.037	0.037	0.290	0.130
	p	0.217	<0.001	0.347	0.352	<0.001	0.001

SMAD3	r	0.085	0.120	0.054	0.200	0.086	0.046
	p	0.030	0.002	0.170	<0.001	0.029	0.246

SMAD4	r	0.130	0.230	0.130	0.084	0.340	0.220
	p	0.001	<0.001	0.001	0.032	<0.001	<0.001

SMAD5	r	−0.062	0.050	−0.150	−0.041	0.110	−0.031
	p	0.116	0.205	<0.001	0.297	0.005	0.436

SMAD6	r	0.004	−0.097	0.089	0.032	−0.310	−0.170
	p	0.910	0.014	0.024	0.417	<0.001	<0.001

SMAD7	r	0.260	0.170	0.160	0.260	0.130	0.078
	p	<0.001	<0.001	<0.001	<0.001	0.001	0.048

SMAD9	r	0.047	0.009	0.071	0.100	−0.033	−0.110
	p	0.234	0.816	0.072	0.010	0.396	0.007

## Data Availability

The data sets used in this study are available from the corresponding and the first author.

## Nomenclature

CRC: Colorectal cancer
SMAD: Small mothers against decapentaplegic
TGF-*β*: Transforming growth factor *β*
ERK: Extracellular regulated protein kinases
MAPK: Mitogen-activated protein kinase
JAK: Janus kinase
STAT: Signal transducer and activator of transcription
BMP: Bone morphogenetic protein
HPA: Human Protein Atlas
GEPIA: Gene expression profiling interactive analysis
TCGA: The Cancer Genome Atlas
GTEx: The Genotype-Tissue Expression database
ssGSEA: Single sample gene enrichment analysis
GSVA: Gene set variation analysis
OS: Overall survival
DSS: Disease-specific survival
PFI: Progression-free interval
DCs: Dendritic cells
HCC: Hepatocellular carcinoma
EMT: Epithelial-mesenchymal transition.

## References

[1] Sung H., Ferlay J., Siegel R. L. (2021). Global cancer statistics 2020: GLOBOCAN estimates of incidence and mortality worldwide for 36 cancers in 185 countries. *CA: A Cancer Journal for Clinicians*.

[2] Biller L. H., Schrag D. (2021). Diagnosis and treatment of metastatic colorectal cancer: a review. *JAMA*.

[3] Beets G. L. (2021). Colorectal cancer immunotherapy: a treatment quantum leap. *British Journal of Surgery*.

[4] Chen J., Chang R. (2022). Association of TGF-*β* canonical signaling-related core genes with aortic aneurysms and aortic dissections. *Frontiers in Pharmacology*.

[5] Liu J., Jin J., Liang T., Feng X. H. (2022). To Ub or not to Ub: a regulatory question in TGF-*β* signaling. *Trends in Biochemical Sciences*.

[6] Luo K. (2017). Signaling cross talk between TGF-*β*/smad and other signaling pathways. *Cold Spring Harbor Perspectives in Biology*.

[7] Guan R., Lin R., Jin R. (2020). Chitinase-like protein YKL-40 regulates human bronchial epithelial cells proliferation, apoptosis, and migration through TGF-*β*1/Smads pathway. *Human and Experimental Toxicology*.

[8] Korkut A., Zaidi S., Kanchi R. S. (2018). A pan-cancer analysis reveals high-frequency genetic alterations in mediators of signaling by the TGF-*β* superfamily. *Cell Syst*.

[9] David C. J., Massagué J. (2018). Contextual determinants of TGF*β* action in development, immunity and cancer. *Nature Reviews Molecular Cell Biology*.

[10] Gough N. R., Xiang X., Mishra L. (2021). TGF-Β signaling in liver, pancreas, and gastrointestinal diseases and cancer. *Gastroenterology*.

[11] Mohammadi-Shemirani P., Sood T., Paré G. (2023). From′omics to multi-omics technologies: the discovery of novel causal mediators. *Current Atherosclerosis Reports*.

[12] Ullah I., Yang L., Yin F. T. (2022). Multi-omics approaches in colorectal cancer screening and diagnosis, recent updates and future perspectives. *Cancers*.

[13] Uhlen M., Zhang C., Lee S. (2017). A pathology atlas of the human cancer transcriptome. *Science*.

[14] Tang Z., Li C., Kang B., Gao G., Li C., Zhang Z. (2017). GEPIA: a web server for cancer and normal gene expression profiling and interactive analyses. *Nucleic Acids Research*.

[15] Unberath P., Knell C., Prokosch H. U., Christoph J. (2019). Developing new analysis functions for a translational research platform: extending the cBioPortal for cancer genomics. *Studies in Health Technology and Informatics*.

[16] Franz M., Rodriguez H., Lopes C. (2018). GeneMANIA update 2018. *Nucleic Acids Research*.

[17] Hänzelmann S., Castelo R., Guinney J. (2013). GSVA: gene set variation analysis for microarray and RNA-seq data. *BMC Bioinformatics*.

[18] Zhang H. W., Guo Y., Sun L. X., Ni F. B., Xu K. (2021). Prognostic value of small mother against decapentaplegic expression in human gastric cancer. *Bioengineered*.

[19] Pan S., Zhou G., Hu W., Pei H. (2020). SMAD-6, -7 and -9 are potential molecular biomarkers for the prognosis in human lung cancer. *Oncology Letters*.

[20] Yang D., Hou T., Li L. (2017). Smad1 promotes colorectal cancer cell migration through Ajuba transactivation. *Oncotarget*.

[21] Zhou J., Wang M., Mao A. (2021). Long noncoding RNA MALAT1 sponging miR-26a-5p to modulate Smad1 contributes to colorectal cancer progression by regulating autophagy. *Carcinogenesis*.

[22] Brochu-Gaudreau K., Charbonneau M., Harper K., Dubois C. M. (2022). Hypoxia selectively increases a SMAD3 signaling Axis to promote cancer cell invasion. *Cancers*.

[23] Lin C., Zhang J., Lu Y. (2018). NIT1 suppresses tumour proliferation by activating the TGF*β*1-Smad2/3 signalling pathway in colorectal cancer. *Cell Death and Disease*.

[24] Zheng X., Chen J., Nan T. (2022). FAM198B promotes colorectal cancer progression by regulating the polarization of tumor-associated macrophages via the SMAD2 signaling pathway. *Bioengineered*.

[25] Song K., Lee H. S., Jia L., Chelakkot C., Rajasekaran N., Shin Y. K. (2022). SMAD4 controls cancer cell metabolism by regulating methylmalonic aciduria cobalamin deficiency (cbl) B type. *Molecular Cell*.

[26] Li H., Liang W., Zhang H., Shui Y., Zhang Z. (2021). *Retracted* : MicroRNA‐4429 restrains colorectal cancer cell invasion and migration via regulating SMAD3‐induced epithelial–mesenchymal transition. *Journal of Cellular Physiology*.

[27] Fang T., Liang T., Wang Y. (2021). Prognostic role and clinicopathological features of SMAD4 gene mutation in colorectal cancer: a systematic review and meta-analysis. *BMC Gastroenterology*.

[28] Yang S., Yao L., Wang X. (2022). Exosomes derived from SW480-resistant colon cancer cells are promote angiogenesis via BMP-2/smad5 signaling pathway. *Applied Bionics and Biomechanics*.

[29] Lu W., Sun J., Zhou H. (2020). HNF1B inhibits cell proliferation via repression of SMAD6 expression in prostate cancer. *Journal of Cellular and Molecular Medicine*.

[30] Bayat Z., Ghaemi Z., Behmanesh M., Soltani B. M. (2021). Hsa-miR-186-5p regulates TGF*β* signaling pathway through expression suppression of SMAD6 and SMAD7 genes in colorectal cancer. *Biological Chemistry*.

[31] Alidoust M., Hamzehzadeh L., Khorshid Shamshiri A. (2022). Association of SMAD7 genetic markers and haplotypes with colorectal cancer risk. *BMC Medical Genomics*.

[32] Li N. (2020). CircTBL1XR1/miR-424 axis regulates Smad7 to promote the proliferation and metastasis of colorectal cancer. *Journal of Gastrointestinal Oncology*.

[33] Zhao S., Sun H., Jiang W. (2017). miR-4775 promotes colorectal cancer invasion and metastasis via the Smad7/TGF*β*-mediated epithelial to mesenchymal transition. *Molecular Cancer*.

[34] Ding N., Luo H., Zhang T., Yao Y., He Y. (2021). *Correlation between SMADs and Colorectal Cancer Expression, Prognosis, and Immune Infiltrates*.

